# Supplementary material for: HDXmodeller: an online webserver for high-resolution HDX-MS with auto-validation
Source: Commun Biol. 2021 Feb 15;4:199. doi: 10.1038/s42003-021-01709-x (PMC7884430; doi:10.1038/s42003-021-01709-x)
Supplement: Supplementary file 2 — Supplementary Information [file 42003_2021_1709_MOESM2_ESM.pdf]

HDXmodeler: a webserver for high-resolution HDX-MS with auto-validation

Supporting information

Supplementary Figure 1

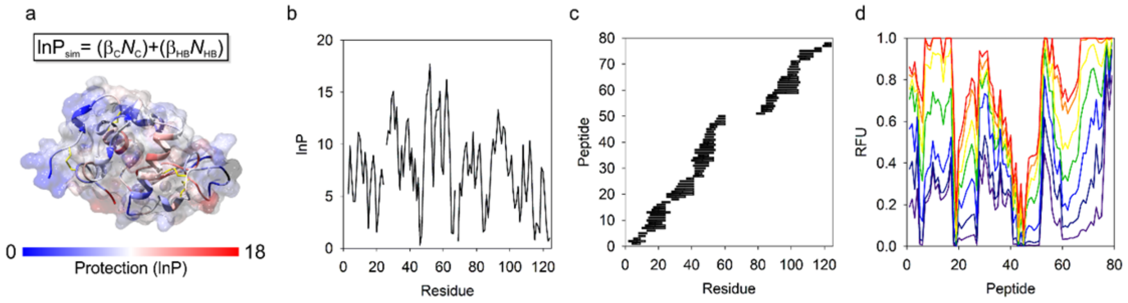

Workflow for the preparation of reference data: (a) lnP values were first simulated directly from protein crystal structures using a well-known expression where protection from solvent exchange in folded proteins is considered as a function of hydrogen bonding and stability calculated from contacting heavy atoms (shown). **(b)** Plot of the simulated lnP values for alpha lactoglobulin, gaps in the projection are from proline residues. **(c)** The simulated lnP were then taken along with the chemical exchange rates to calculate the observed exchange rates for each amino acid (Equation 1). From this value the isotope uptake rate of each amino acid could be determined, and these were projected onto an experimental peptide map. **(d)** Simulated HDX-MS data were then prepared reporting the RFU of each peptide over 7 different time points from 15 seconds to 8 hours. The simulated HDX-MS data were then submitted to HDXmodeller for optimisation and the performance of the optimisation determined by direct comparison of the modelled and simulated lnP.

Supplementary Figure 2

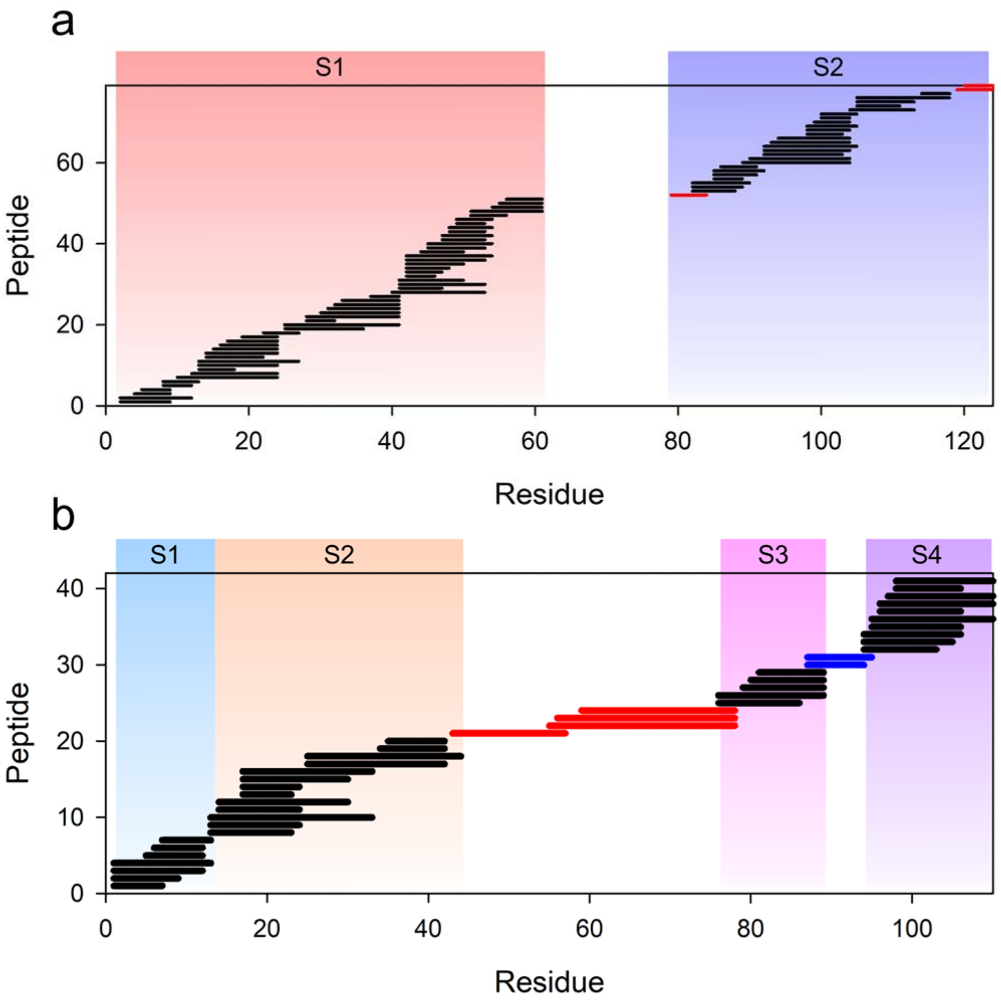

Example peptide maps: Peptide maps associated with the example HDXmodeller outputs shown in Figure 1 for alpha lactalbumin **(a)** and barnase **(b)**. Plots are also related to the peptide occupancy data shown in Figures 1h – i. Data subsections that were optimised separately are coloured and denoted (S). Red bars denote peptides with occupancy scores below the 2.5 cut-off which were deleted, blues bars represent bridging peptides eliminated from the barnase dataset in order to create subsections 3 and 4.

Supplementary Figure 3

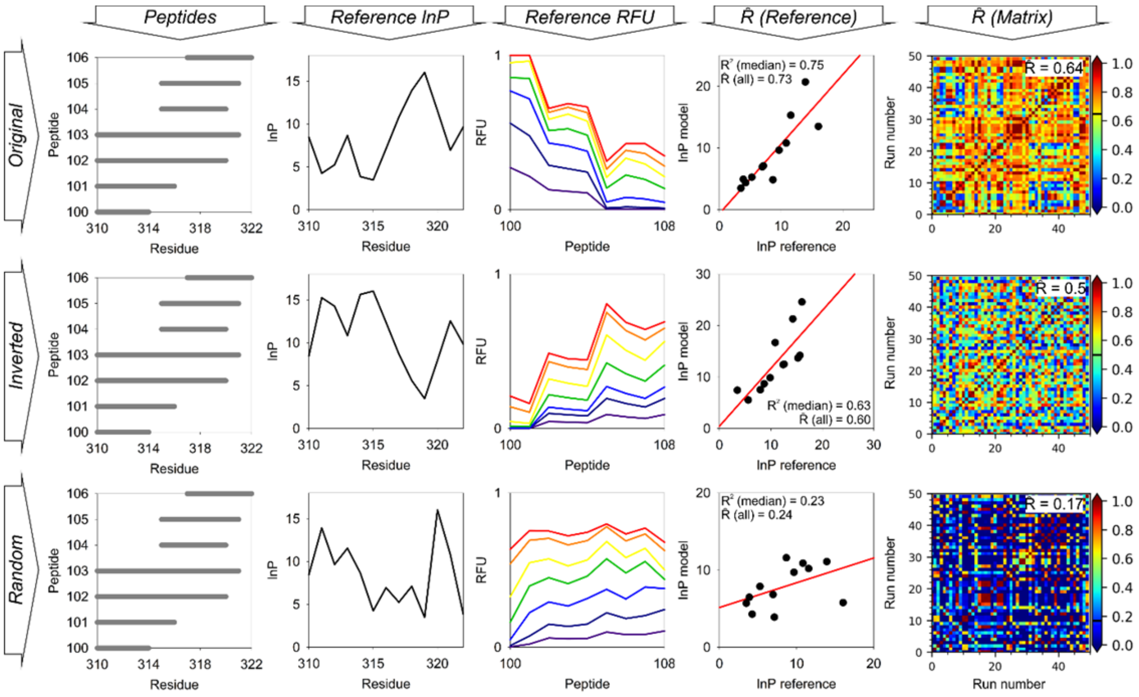

Effect of exchange rates on modelling outcomes: Three different datasets are shown, original **(top row)**, inverted **(middle row)** and random **(bottom row)**. Each dataset was built using an identical peptide map spanning 7 peptides and 13 amino acids, the amino-terminus residue is not shown. For each dataset three different sets of reference lnP values were simulated and used to project the relative fractional uptake (RFU) of each peptide (Online methods). RFU data were then subjected to optimisation by HDXmodeller and the modelled lnP values compared with the reference lnP to obtain the median R^2 and $\hat{R}$ -reference as shown. The auto-validation matrix for each optimisation is also shown along with the $\hat{R}$-matrix score. The plot shows the dependence of optimisation outcomes on the RFU.

Supplementary Figure 4

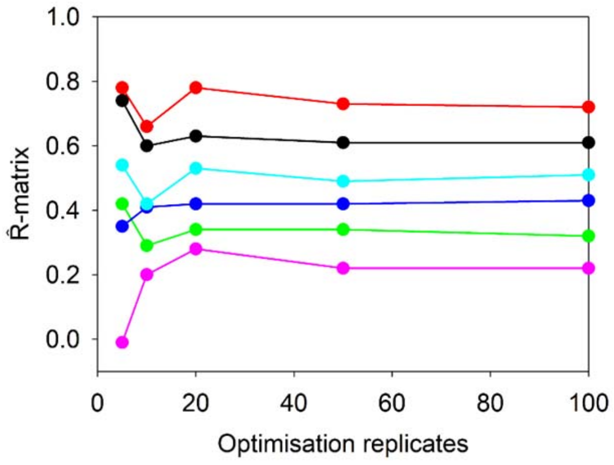

R̂-matrix scores as a function of optimisation replicates: Trends are given for 6 different reference datasets showing R̂-matrix values as a function of the number of optimisation replicates. Plot demonstrates no benefit in optimisation (R̂-matrix) between 50 and 100 replicate runs and that 10 replicates are sufficient to predict these values.

Supplementary Figure 5

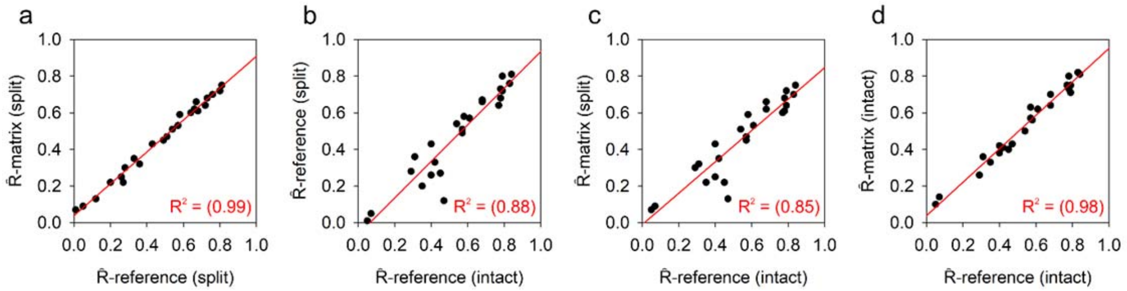

Comparison of HDXmodeller outcomes for different data input strategies: (a) relationship between $\hat{R}$-reference and $\hat{R}$-matrix scores for HDX-MS reference data optimised as independent subsections (as shown in the main paper). **(b)** relationship between $\hat{R}$-reference values for HDX-MS data where independent subsections of protein data were either submitted as separate jobs (split) or combined and submitted as a single file (intact). The lack of a perfect correlation between these data reflects the changes in optimisation outcomes depending on the input strategy. **(c)** plot of the relationship between $\hat{R}$-reference (intact) and $\hat{R}$-matrix (split). Plot demonstrates the inability of these $\hat{R}$-matrix values to accurately predict the $\hat{R}$-reference scores, as expected. **(d)** use of stand-alone matrix optimiser to calculate $\hat{R}$-matrix from combined subsections, note the recovery of $\hat{R}$-matrix to accurately predict the $\hat{R}$-reference values.
